# Rein tensions and behaviour with five rein types in international-level vaulting horses

**DOI:** 10.1371/journal.pone.0311919

**Published:** 2024-10-17

**Authors:** Sophie Biau, Elena Pycik, Laetitia Boichot, Lise-Charlotte Berg, Alice Ruet

**Affiliations:** 1 IFCE (French Horse and Riding Institute), Saumur Technical Platform, Saumur, France; 2 Department of Veterinary Clinical Sciences, University of Copenhagen, Taastrup, Denmark; 3 INRAe, CNRS, University of Tours, PRC, Nouzilly, France; Massey University, NEW ZEALAND

## Abstract

Health and performance of vaulting horses cantering with reins might be affected by rein tensions. The primary aim of this present study was to measure rein and lunge line tensions in international-level vaulting horses with several types of reins adjusted in accordance with the requirements of the FEI Vaulting Rules and study the effect of reins types on it. The secondary aim was to evaluate behavioural signs of discomfort under the same conditions and study the effect of reins types on it. The relationship between tensions and behavioural signs of discomfort was then explored. Thirty-nine international-level vaulting horses took part in this study and tested side reins either with an elastic part, all elastic or without elastic, draw reins with an upper adjustment triangle and side reins fixed on the noseband. Tensions of the left and right reins and the lunge line were measured with sensors at a rate of 80 Hz for a canter of 1min30 on a 15-meter circle, without a vaulter, and behavioural analyses (on the locomotion, tail, head, mouth and ears movements) were performed using video filmed by a camera attached to the lunger’s chest. Mixed models revealed that the Root mean square (RMS) of the tensions of the left and right reins, of the total, as well as the lunge line, were significantly influenced by rein type (p<0.001) in all cases). Total RMS tension ranged from 6.2 kg to 11.7 kg with a right RMS tension superior to left RMS (p < 0.001). When tension was high, mouth opening increased (p = 0.007). It was with draw reins that total tension (p < 0.05) and mouth openings were lower (p = 0.005), even if a high proportion of variance of these discomfort behaviours could be explained by the individual horse.

## Introduction

Equestrian sports involve physical contact between humans and horses using purpose-specific equipment to control speed, direction, and locomotion, whether by a rider or by a lunger. In the case of the vaulting horse, the contact between horse and human is via the lunge line and the horse is reined. The equestrian discipline of vaulting requires the use of reins to encourage an outline and body position from the horse that supports the "bridge of engagement", an energetic bridge between the hindquarters and the front to enhance the horse’s ability to carry the vaulter and allow the vaulter to perform. The current FEI Vaulting Rules (2023) [1] require the use of side reins during tests and allow the use of draw reins during warm-up. The FEI Vaulting Guidelines (2023) [2] state that the connection on the side reins must be soft, and that with the side reins properly adjusted, the horse can carry its nose correctly on or slightly in front of the vertical. They are used to modify the position of the head/neck angle in order to achieve the ideal canter described for vaulting, because it is known that there is a link between the position of the head/neck p and locomotion: the position of the head and neck significantly influences the kinematics of the horse [3, 4]. While it is accepted that regular contact is essential to develop the "connection", the optimal degree of tension remains to be defined. Studies have evaluated the tension applied to the horse’s mouth and linked it to health, behaviour, performance, and learning. Oral injuries are common in ridden horses [5–9] and increase with the level of competition [6]. High tensions increase pressure on the oral tissues [10–12] and are associated with several types of pathology. Bit-related injuries due to excessive rein tension include ulcerations of tissues lining the mouth, lacerations of the lips and tongue, and jaw fractures [10, 13–15]. When tensions are high, horses may show signs of discomfort such as headshaking, opening of the mouth [16], tail swishing [16–18]. In performance tests for young ridden horses, rideability scores decrease when rein tension increases, and a lower rideability score was linked to more discomfort behaviours, such as, for example shaking of the head, opening the mouth or tail swishing [11]. These behaviours are characterized by horses having difficulty coping with mental or physical discomfort and are not seen in free-roaming wild horses [19]. However, they are characteristic of ridden horses [15]. In addition, reins and bit use is based on negative reinforcement. If the principle of negative reinforcement is not respected, for example if the rider does not reinforce the correct behaviour by releasing the tension, this leads to a phenomenon of habituation to high tension and the horse is ridden with ever higher tensions [20, 21].

It is therefore relevant to identify rein types that help to reduce the intensity of rein tensions.

Rein tension of an ridden horse is not constant, but follows an oscillatory pattern, depending on the stride events. It was assumed that at canter, a distinct peak occur in the support phase of the diagonal pair of limbs, which was surrounded by two smaller peaks [22]. Rein tensions at canter range from 1.5 to 104 Newton, and peaks of up to 150 N have been reported [18, 21]. These values differ according to the method used to calculate average tensions. Rein tension has mainly been studied using average values calculated over several strides and using the average of maximum values [7, 22–25]. The oscillatory nature of the signal means that it is necessary to calculate a variable reflecting the peaks and troughs of the tension, as well as their duration. Calculating the area under the curve would therefore be relevant [23]. When assessing the impact on oral tissues, it is important to consider both magnitude and frequency.

With ridden horses, several factors are known to have an impact on rein tension: gait, discipline, rider’s position, horse, and their combined level of practice, such as the rider’s ability to follow the horse’s movements [11, 22–26]. Equipment can also have an impact on the intensity of tension. Reins and bit types are known to influence applied tension and pressure [7, 8, 11, 27]. Although the presence of elastic on the reins has not been shown to reduce the number of discomfort behaviours in horses ridden by beginners, it has been demonstrated to reduce tension peaks in unridden horses. It is also known that rein length affects tension [23].

The assessment of discomfort behaviours is a valid method for determining responses in horses to stress, fear and anxiety [18, 28, 29]. It has been suggested that, in exercising horses, behaviour may be a more reliable indicator of stress than physiological measures that are influenced by exercise [28]. Several behaviours can also be an expression of pain [15].

The primary aim of this present study was to measure reins and lunge line tensions in international-level vaulting horses with several types of reins adjusted in accordance with the requirements of the FEI Vaulting Rules, and study the effect of reins types on it [1]. The secondary aim was to evaluate behavioural signs of discomfort under the same conditions, study the effect of reins types on it, and link them to tensions.

The reins tested were those used in competition. The vaulting horse in competition is equipped with side reins, and it can also be equipped with draw reins but only during warm-up before the test [2]. In light of the present literature, rein types other than the standard ones used in the competition were also tested. A no-contact mouth rein was included, as well as a fully elastic rein. By introducing a possible mobility of the head with draw reins and elastic side reins, we expected lower tensions with draw reins than with side reins and consequently fewer signs of discomfort expressed; and by eliminating the tension on the mouth, we expected fewer signs of discomfort with the rein with no contact with the mouth.

The results of this study should make it possible to identify the rein type with which tensions are lowest and consequently help to minimize signs of discomfort in the vaulting horse in a sporting context. The Fédération Equestre Internationale (FEI) already has rules to protect the health and welfare of horses during competitions, however, information is needed regarding the use of reins and their impact on the horse’s comfort to guide the development of rules to protect the horse.

## Materials and methods

### Animals

A total of 39 high-level vaulting horses (13 ± 4 years old with 4 ± 3 years of international experience; mean ± SD) were tested in the present study. All horses had previously competed at international level. There were 35 geldings, two mares and two stallions. They were lunged by 19 lungers, known to the horses, with 9 ± 4 years of international experience.

### Study design

The study was carried out between 12–2021 and 02–2023 as a multicentre study at seven different test centres in four countries (France, the Netherlands, Denmark and Finland). For each horse, the tests were performed on two consecutive days in an indoor arena on a soft, resilient surface. The test area was enclosed by a low fence or other suitable material to give a diameter of 22 metres with the lunger in the centre. All tests were recorded with a camera (Go pro Hero 8, GoPro, Inc. 3025 Clearview Way, San Mateo, CA 94402, États-Unis) attached to the front of the lunger. All horses were wearing their regular equipment used for vaulting except for side reins. They were also fitted with an inertial measuring unit (IMU) (Opal sensor, 6g, APDM Wearable Technologies Inc. 7204 SW Durham Road, Suite 800 (Bldg. Q), Portland, OR 97224, Etats-Unis) attached to the surcingle against the sternum. The sensors include 3 axis accelerometer, a 3 axis gyro, a 3 axis magnetometer, stride frequency was 128 Hz and accelerometers have been configured in a high 6G mode.

At the beginning of the test, the horse would arrive in the arena warmed up. For each rein type, before starting the recording the horse/lunger combination had one minute to acclimatize to the rein type. Then a duration of 1 minute 30 sec of left canter was recorded. The horse worked on a circle of 14 ± 1 m in diameter. The mean speed of the left canter was 4± 0.5 m/s, and the mean stride frequency was 1.6 ± 0.1 stride/s. Between each rein type, the horses’ mouths were checked for lesions, and the horses were hand walked for five minutes without side reins.

**Fig 1:**
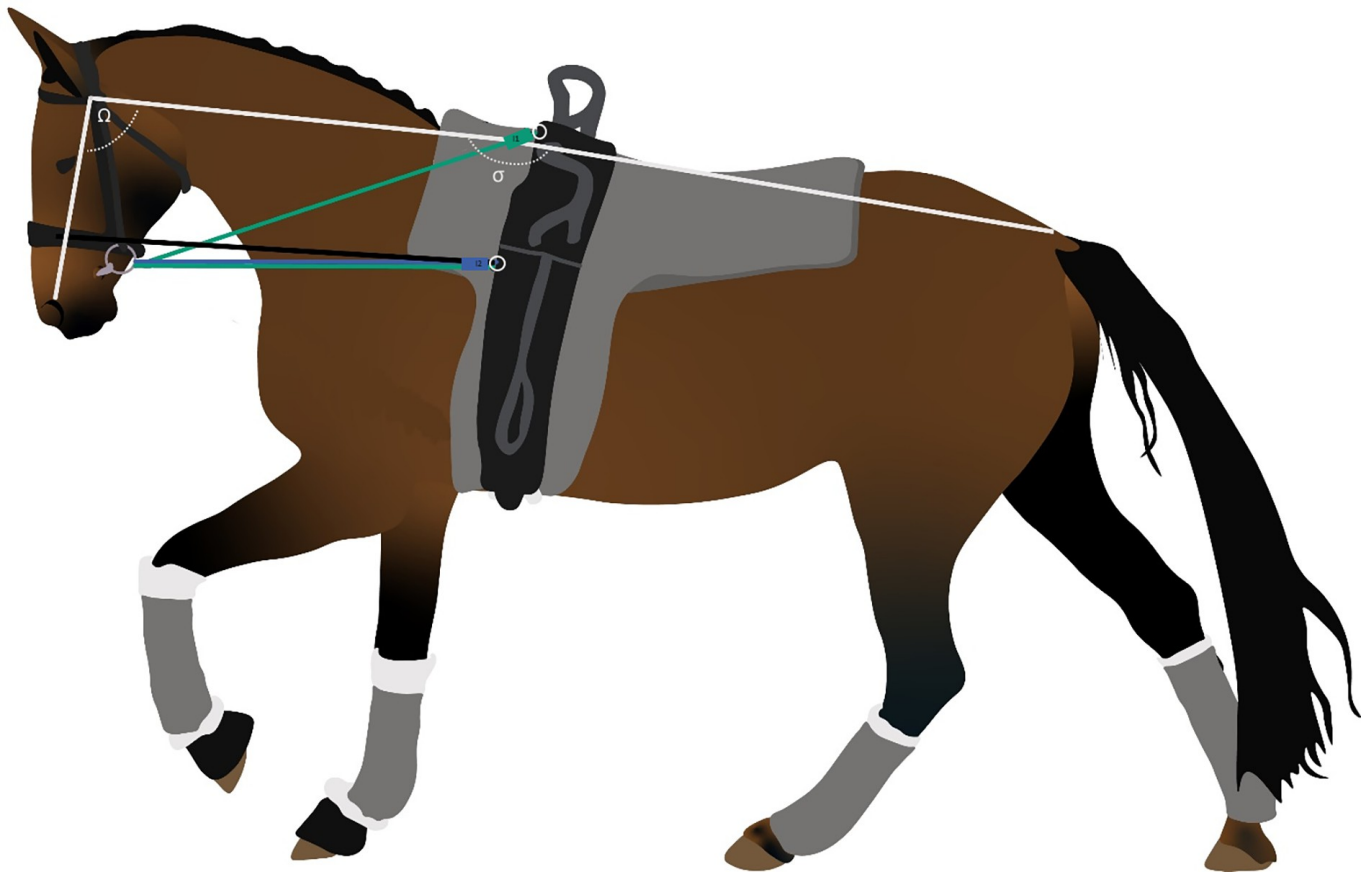
Reins diagram. Side reins in blue, upper triangle of the draw reins in green, and the side reins fixed on the noseband (NOM) in black. The adjustment of reins was such that the head and neck orientation imposed a head-neck angle (Ω) of 92° ± 10 and a neck-trunk angle (σ) of 178° ± 11. As described in the FEI rules, the nose was in front of the vertical axis at 7° ± 6. For the side rein types (blue rectangle I2) the sensor was attached between the extremity of the rein and the surcingle at the median attachment point. For the draw rein, the sensor (green rectangle I1) was attached between the extremity of the rein and the surcingle at the dorsal attachment point.

All horses were tested with the five types of reins in a randomized design with three or two types tested on the first day and two or three types tested on the second day. The tested rein types included three types of side reins, one type of draw reins, and one type of side reins without contact with the mouth (Fig 1):

- All-leather side reins: "*Without elastic*"; This is a standard rein type for competitions. It joins the bit ring and the surcingle. It is made entirely of leather
- Side reins with a small part of elastic: "*With elastic*"; this is a standard rein type for competitions. It joins the bit ring and the surcingle. It is made of leather with an elastic piece measuring 6 cm.
- All-elastic side reins: "*All-elastic*"; this is not accepted in competition It joins the bit ring and the surcingle. It is completely elastic.
- Draw reins adjusted to a small upper adjustment triangle. They were attached to the dorsal side of the surcingle and ran through the bit back to the lateral side of the surcingle: "*Upper triangle*". This is a rein type that is only accepted for warming up competitions. It connects two points of attachment of the surcingle and runs through the ring of the bit. The adjustment was such that the average angle (the top fixing point on the surcingle, the ring bit and the bottom fixing point on the surcingle) was 32° ± 5.
- Side reins without direct contact to the mouth: "*Not on the mouth"* or *"NOM”*. This is a non-standard rein type, not accepted in competition. Side leather reins connect the surcingle and a lateral attachment point at the noseband.

The lunge line was attached to the ring bit whatever the rein type.

Six horses were not tested with the "*Not on the mouth*" side reins. This rein type was not commonly used on some of the horses and was therefore optional. Six lungers opted out of this rein type. The reins were adjusted in accordance with the FEI’s Vaulting Rules (“With the side reins properly adjusted, the horse can carry its nose correctly on or slightly in front of the vertical.”). In order to allow the horse to bend on the circle, the lungers would typically adjust the inside rein to be slightly shorter than the outside rein. This difference in length between the two sides was left at the discretion of the lunger. The draw reins were typically two to 2.5 times longer than the side reins. Validation of the proper adjustment was carried out retrospectively (Fig 1). The head and neck positions showed head-neck angle (Fig 1, Ω) of 92° ± 10 and neck-trunk angle (Fig 1, σ) of 178° ± 11. These values were measured from the zygomatic arch, the withers, and base of the tail (white line) taken from still images from the camera worn by the lunger, for a canter stride. The verticality of the nose was checked with an IMU (Opal, APDM Inc., Portland, OR, USA) attached to the snaffle for the canters of 22 horses; as described in the FEI rules, the nose was in front of the vertical axis at 7° ± 6.

### Ethical statement

Behavioural research is approved by the FEI (Vaulting) and didn’t require study-specific permission. The behavioural observations of the horses were carried out in compliance with the ethical guidelines of the International Society for Applied Ethology. This research was non-invasive, and the test setup corresponded to a regular training session. The reins tested are part of the horses’ normal equipment. The tension was measured with a sensor integrated into the reins that the horses usually use. Chief trainers of the testing stations and owners, lungers and vaulters volunteered their horses to participate in the study. They all signed an informed consent form beforehand. If the participant was a minor, one of the two parents signed. No personal data was collected. The age and number of years of practice were collected from the FEI website (https://www.fei.org/vaulting/athletes). Data has been anonymized for analysis.

#### Mouth examination

An examination of the horse’s mouth before and after each test was carried out by an qualified FEI veterinarian equipped with disposable gloves. The examination consisted first of all in palpating the upper and lower bars, looking for sensitivity or injuries, and secondly, with the mouth open and the tongue held in place, to check for injuries to bars of the mandibule, the lips, corners and tongue. These examinations ensured respect for the physical integrity of the horse and the quality of the data collection. A grid was filled in for each test and the results were communicated live. No injuries were reported during this study.

### Data collection

#### Rein and lunge line tensions

Left and right rein tensions were measured with sensors (IPOS Technology B.V., 5656 AE Eindhoven, the Netherlands, https://www.linkedin.com/company/ipos-technology/about/). For the side rein types, the left sensor was attached between the extremity of the left rein and the surcingle at the median attachment point. The right sensor was attached between the end of the right rein and the surcingle at the median attachment point (Fig 1). The length of the sensor (1.16 cm, <100g) was subtracted from the usual length of the side rein. For the draw rein, the left sensor was attached between the extremity of the left rein and the surcingle at the right dorsal attachment point. The right sensor was attached between the extremity of the left rein and the surcingle at the right dorsal attachment point (Fig 1). The angle between the dorsal attachment point of the surcingle, the ring of the bit and the median attachment point of the surcingle was 32° ± 5. It was calculated from the lunger’s camera, always placed at the same distance and perpendicular to the horse. The analysis was carried out using Kinovea® software. For each horse canter with a draw rein, 3 measurements at 3 stride events were taken.

The tension measurement range was specified by the manufacturer to be between 0 N and 500 N. Data were sampled at a rate of 80 Hz. Measurements were received wirelessly (via the IPOS app) by a smartphone worn by the lunger via Bluetooth (minimum required version 4.3). Using the app, the rein sensors were calibrated (zero offsets adjusted) before each test by placing the sensors on a horizontal surface with no load applied. Tensions, expressed in kg were recorded continuously throughout the test. The data was then retrieved and processed using Matlab R2019b software (MathWorks®) v. 2019b. The signal was cut according to the times recorded on the video from the camera fixed to the lunger who filmed the test. The film and sensors were systematically synchronized for each test (three strong pulls were filmed on the sensor before and after each test).

In order to illustrate the mean tension of this periodic signal, the calculation of the square root of the arithmetic mean of the squares of the values, called Root mean square (RMS), was chosen. This mean was calculated for the left and right tensions (“LEFT”: RMS of the left rein tension; “RIGHT”: RMS of the right rein tension) and for the sum of the two rein tensions (“TOTAL”: RMS of the left and right rein tension). In addition, to quantify a possible difference in tension between the left and right reins, the value of the ratio between the two tensions (RIGHT/LEFT) was calculated. This represented the asymmetry coefficient (“ASYMMETRY”). The higher the coefficient, the greater the difference between the right and left tension. Perfect symmetry between the right and the left corresponds to a coefficient of asymmetry = Specially in the case of the draw reins, the tension measured on the left and the tension measured on the right were multiplied by a coefficient depending on the angle. Assuming that the reins were taut, the resulting force at the bit ring was equal to the forces at the surcingle attachment points and with an angle at 32° ± 5, the tension measured by the sensor was multiplied by 1.9 (Data in S2 File). This has been done for the tension of the right sensor and for the tension of the left sensor. Lunge line tension was measured using the same equipment as for rein tensions: the lunge line attached to the sensor, which was attached to the left bit ring. As with the reins, the tension of the lunge line is not constant. For each stride, the contact reaches a maximum value and then decreases to a minimum value. Depending on the lunger and the horse, there may be one or two additional peaks of varying magnitude. To illustrate the mean tension of this periodic signal, the calculation of the RMS was also chosen (“LUNGE”: RMS of the lunge line tension). It has been calculated for each rein type.

#### Behaviour

Horses were filmed using GoPro® camera attached to a harness to the front of the lunger’s chest. Behavioural analyses were performed using Boris© [30] by an experienced observer. The recorded behaviours are described in Table 1. Two different sampling methods were used to analyse the behaviour of the horses. LOC, TAIL, HEAD and MOUTH behaviours were recorded by sampling each time they were expressed during each test with the different reins.

**Table 1:** The six behaviours studied for the 39 vaulting horses.

Behaviour	Description
LOC	Breaking the gait (i.e., the horse spontaneously switches from canter to trot) counter canter (i.e., the horse is cantering on one lead while curving in the opposite direction). Shy (i.e., the horse shows a startle reaction and attempts to flee). Kick and buck (i.e., the horse expresses undesirable movement with front or hindlegs). Rear (i.e., the horse raises its forelegs off the ground). Hollowing of the back (i.e., the horse shows a quick and brief raising of the head).
TAIL	Tail swishing (i.e., the horse shows circular, dorsoventral or lateral movement of the tail.
HEAD	Head tossing (i.e., the horse shows repetitive fast up and down motion of the head). Headshaking (i.e., the horse expresses repetitive head movements from the right to the left). Pulling the reins (i.e., the horse shows fast forward motion of the head).
MOUTH	Opening the mouth (i.e., the horse clearly opens the mouth and upper and lower teeth may be visible). Sticking the tongue out (i.e., the horse moves the tongue in and out of the mouth).
EARS	Ears backwards: the horse rotates both ears caudally with ear hole pointing outwards.
ABN TAIL	Abnormal position of the tail: The horse has its tail clamped to the middle, a raised tail carriage or a crooked tail.

As the duration of 1 min 30 sec was not always perfectly followed (with a loss or gain of a few seconds), the frequencies of expression of each of the behaviours were then calculated over a standardised period of 1 minute.

EARS and ABN TAIL behaviours were recorded using the scan sampling method, which consisted of recording the absence or presence of ears backwards and abnormal tail positions every five seconds. Percentages of the presence of the two behaviours were then computed from the total number of scans recorded. The observer watched the videos as many times as necessary to identify all the behaviours at real speed.

### Statistical analyses

The statistical analysis was carried out with 189 records: 5 records per horse corresponding to the 5 rein types, with the exception of the NOTM which was not tested by 4 horses and the 1 horse which did not have the side reins without elastic. Statistical processing was performed with 173 recordings for LOC, TAIL and HEAD, 170 for MOUTH, 175 for EARS and ABN Tail, 164 for TOTAL and 156 for LUNGE, due to lack of visibility of the signs of discomfort in some videos and technical problems in recording tensions. Mixed models were used to test the effect of rein type on rein and lunge line tensions, as well as on each behaviour separately. Thus, eleven different models were built with LEFT (kg), RIGHT(kg), TOTAL(kg), ASYMMETRY, LUNGE(kg), LOC (number/1 min), TAIL (number/1 min), HEAD (number/1 min), MOUTH (number/1 min), EARS (% of presence out of the total number of scans recorded), and ABN TAIL (% of presence out of the total number of scans recorded), as continuous outcome variables. Rein type was considered as a fixed-effect parameter and a “horse” effect was included as a random factor to account for individual variation in paired behavioural measures, as all the horses were tested with each rein type. More precisely, LEFT, RIGHT, TOTAL, ASYMMETRY, LUNGE, LOC, MOUTH and ABN TAIL were analysed using linear mixed-effects models (LMMs; lmer function, lme4 R library) after square root or log transformation of the response variable if needed. These models were fitted with restricted maximum likelihood. P-values were computed using the Satterthwaite approximation (anova function, lmerTest R library). TAIL, HEAD and EARS were analyzed using generalized linear mixed models with Tweedie distributions, because of the high number of null values in these behaviours (GLMMs; glmmTMB function, glmmTMB R library).

All the residuals were checked graphically for normal distribution and homoscedasticity (simulateResiduals function, DHARMa R library). Conditional r^2^, marginal r^2^ and ICC (ie used to obtain an estimate of the intra-individual correlation between the different tests) were calculated using performance function from the performance R library. The ICC was high if it exceeded 0.70, and moderate if it lay between 0.40 and 0.70 [31]. When appropriate, Tukey post-hoc tests (glht function, multcomp R library) were carried out to further investigate the significant effects of rein type.

Spearman’s correlation analyses were used to determine any relationship between LEFT, RIGHT, TOTAL, ASYMMETRY and the behaviours.

All statistical analyses were performed using R software (version 2023.12.1, R Development Core Team, Vienna, Austria, 2019) with a significance level of (p) ≤ 0.05.

## Results

Table 2 shows the descriptive statistics of all the variables recorded (RMS reins and lunge line tensions expressed in Kg as well as behaviours, expressed in number of behaviours per/one minute of canter and Percentages of presence out of the total number of scans recorded (one scan every five seconds) for each rein type tested.

**Table 2:** Rein and lunge line tensions, as well as behaviours for each rein type.

Variables	With elastic	All elastic	Without elastic	Draw rein	Not on the mouth
*Rein tension (kg)*					
**LEFT**	3.1±1.7^a^ (1.7,2.6,4.4)	2.9±1.6^a^ (1.4,2.9,3.6)	3.0±1.7^a^ (1.6,2.6,3.9)	2.4±1.3^b^ (1.2,2.1,3.5)	5.8±2.5^c^ (3.5,5.5,7.3)
**RIGHT**	4.4±2.0^a^ (2.5,4.3,5.8)	4.0±1.7^a^ (2.7,3.8,5.2)	4.6±2.1^a^ (3.3,4.1,5.7)	3.8±1.7^a^ (2.6,3.5,5.1)	5.8±2.4^b^ (3.8,5.9,7.0)
**TOTAL**	7.4 ±3.6^a^ (4.6,6.4,9.9)	6.9±3.0^ab^ (4.2,6.8,9.0)	7.6±3.4^a^ (4.9,6.9,10.2)	6.2±2.6^b^ (3.9,6.5,7.9)	11.7±4.7^c^ (7.4,11.3,12.9)
**ASYMMETRY**	1.6±0.6^a^ (1.2,1.3,1.8)	1.6±0.9^a^ (1.1,1.3,1.8)	1.8±1.7^a^ (1.2,1.4,1.9)	1.9±1.0^a^ (1.2,1.7,2.2)	1.1±0.3^b^ (0.9,1.0,1.1)
*Lunge line tension (kg)*					
**LUNGE**	3.3±1.9^a^ (1.7,3.3,4.2)	4.1±2.4^a^ (2.3,3.6,4.7)	3.9±2.2^a^ (2.2,3.4,4.6)	3.6±2.2^a^ (2.2,3.7,4.1)	2.8±2.2^ab^ (1.6,2.4,2.9)
*Behaviour*					
LOC^1^	1.1±1.2 (0, 0.9, 1.7)	1.6±2.5 (0, 1.1, 1.9)	1.8±2.1 (0, 0.9, 2.7)	2.2±2.9 (0, 1.1, 2.3)	1.9±2.8 (0, 0.9, 2.4)
TAIL^1^	0.7±1.3 (0, 0, 0.9)	1.1±2.2 (0, 0, 1.3)	1.0±2.6 (0, 0, 0.8)	0.7±1.3 (0, 0, 0.9)	0.6±1.6 (0, 0, 0)
HEAD^1^	1.3±5.9^ab^ (0, 0, 0)	2.8±8.8^a^ (0, 0, 0.9)	0.6±1.9^ab^ (0, 0, 0)	0.3±1.0^b^ (0, 0, 0)	0.6±2.0^ab^ (0, 0, 0)
MOUTH^1^	22.6±25.1^ab^ (2.8, 11.2, 36.0)	23.5±24.2^ab^ (4.7, 14.8, 36.8)	29.4±30.4^a^ (6.3, 13.6, 53.6)	15.5±19.6^b^ (2.7, 9.4, 19.6)	17.4±20.0^ab^ (2.7, 10.5, 27.2)
EARS^2^	13.0±16.7 (0, 0, 20.0)	13.2±18.2 (0, 0, 19.4)	11.7±14.9 (0, 0, 25.0)	9.12±14.2 (0, 0, 14.9)	10.7±16.6 (0, 0, 17.0)
ABN TAIL^2^	65.8±35.8 (42.9, 80.0, 100.0)	58.9±31.9 (41.5, 62.5, 83.3)	68.4±29.4 (57.1, 71.7, 90.5)	58.9±40.3 (15.6, 62.5, 100.0)	64.1±34.0 (42.9, 71.4, 100.0)

Mean±SD and quartiles 25th, 50th and 75th are presented (in brackets) of RMS (kg) for tensions and of number of behaviours per min or percentages of canter. Horses were tested with rein types in a random order, at left canter and without the vaulter. N = 39. Variables in bold were significantly influenced by rein type. Letters represent multiple comparison results using Tukey’s post-hoc tests.

^1^Number of behaviours per minute of canter

^2^Percentages of presence out of the total number of scans recorded (one scan every five seconds).

### Rein tension

LMMs revealed that the RMS of the left rein tension, the RMS of the right rein tension, the RMS of the left and right rein tensions, and the asymmetry coefficient were all significantly influenced by rein type. Post-hoc tests revealed that the lowest tension was for the draw reins set to a upper triangle (LEFT: 2.4 ± 1.3 kg; -12.5<Z value<-2.6, p<0.05 in all cases; RIGHT: 3.8 ± 1.7 kg, -6.3<Z value<-1.2 although not statistically different from with elastic, all elastic and without elastic reins; TOTAL: 6.2 ± 2.6 kg, -10.6<Z value<-2.9, p<0.05 in all cases except with all elastic reins; Table 2). In contrast, the highest RMS was for the side reins without direct contact with the mouth (LEFT: 5.8 ± 2.5 kg, 9.7 < Z value < 12.5, p < 0.001 in all cases; RIGHT: 5.8 ± 2.4 kg, 4.0 < Z value < 6.3, p < 0.001 in all cases; TOTAL: 11.7 ±4.7 kg, 7.8 < Z value <10.6, p<0.001 in all cases; Table 2). The asymmetry coefficient was the lowest for the side reins without direct contact with the mouth (1.1 ± 0.3, -6.3< Z value < -4.5, p < 0.001 in all cases; Table 2). The models overall explained respectively 77%, 67%, 76% and 52% of the total variance of LEFT, RIGHT, TOTAL and ASYMMETRY variables, including 27%, 10%, 20% and 14% explained by rein type. The ICCs showed that the overall variation in the rein tension was also moderately explained by the individual horse (i.e. ICC values ranging from 0.40 to 0.70) [31] (Table 3).

**Table 3:** Summary of the results of the models on each of the four rein tensions (Rein tensions ~ type of reins + (1|horse), one model per outcome variable).

Rein tensions	Model type	Family	Transformation	F value	Degrees of freedom	p-value	Conditional R^2^	ICC
							Marginal R^2^	
LEFT	LMM	Gaussian	Square root	43.7	4	<0.001	0.77	0.68
							0.27	
RIGHT	LMM	Gaussian	Square root	11.3	4	<0.001	0.67	0.63
							0.10	
TOTAL	LMM	Gaussian	Square root	31.2	4	<0.001	0.76	0.70
							0.20	
ASYMMETRY	LMM	Gaussian	Log	10.9	4	<0.001	0.52	0.44
							0.14	

“LMM”: linear mixed-effects models *(Tables in S1 File)*

### Lunge line tension

The LMM showed that the RMS of lunge line tension was also significantly influenced by rein type (Table 4). Post-hoc tests revealed that the lowest RMS was for the side reins without direct contact to the mouth (2.8 ± 2.2 kg, -3.9 < Z value < -3.2, 0.003 < p < 0.01; Table 1). Overall, the model explained 76% of the total variance of the LUNGE variable, but only 3% was explained by rein type. The individual horse seems to provide the main explanation for the variation in this lunge line tension (ie the value of the ICC is greater than 0.70; Table 3).

**Table 4:** Summary of the results of the models on the lunge line tension (Lunge line tension ~ type of reins + (1|horse)).

Lunge line tension	Model type	Family	Transformation	F value	Degrees of freedom	p-value	Conditional R^2^	ICC
							Marginal R^2^	
LUNGE	LMM	Gaussian	Square root	5.2	4	<0.001	0.76	0.75
							0.03	

“LMM”: linear mixed-effects models *(Tables in S1 File)*

### Behaviour

LMMs and GLMMs revealed that only the HEAD and MOUTH behaviours were significantly influenced by rein type (Table 4). Post-hoc tests revealed that the mean number of behaviours related to the movements of the neck and head was lower for the draw reins set to an upper triangle compared to the all-elastic side reins (Z = -1.9, p = 0.008; Table 1). The mean number of behaviours related to the mouth was lower for the draw reins set to a upper triangle compared to the without elastic side reins (Z = -1.3, p = 0.005; Table 1). The models explained 71% and 66% of the total variance of HEAD and MOUTH respectively, but only 4% and 3% were explained by rein type. The ICCs showed that a moderate proportion of variance of these two behaviours could be explained by the individual horse (ie, ICC values ranging from 0.40 to 0.70; Table 5).

**Table 5:** Summary of the results of the models on the behaviours.

Behaviour	Model type	Family	Transformation	Test value	Degrees of freedom	p-value	Conditional R^2^	ICC
							Marginal R^2^	
LOC	LMM	Gaussian	Square root	0.9^1^	4	0.46	0.35	0.34
							0.01	
TAIL	GLMM	Tweedie	No transformation	5.4^2^	4	0.25	0.72	0.72
							0.01	
HEAD	GLMM	Tweedie	No transformation	14.8^2^	4	<0.01	0.71	0.69
							0.04	
MOUTH	LMM	Gaussian	Square root	3.6^1^	4	<0.01	0.66	0.65
							0.03	
EARS	GLMM	Tweedie	No transformation	2.0^2^	4	0.73	0.27	0.26
							0.01	
ABN TAIL	LMM	Gaussian	No transformation	0.7^1^	4	0.57	0.45	0.44
							0.01	

(Behaviour ~ type of reins + (1|horse), one model per outcome variable).

“LMM”: linear mixed-effects models. “GLMM”: generalized linear mixed models *(Tables in S1 File).*

^1^F value. ^2^Χ^2^ value

LOC, TAIL, EARS and ABN TAIL were not significantly influenced by rein type. The overall variation in tail swishing and abnormal position of the tail was moderately to highly explained by the individual horse (ie the ICC values being greater than 0.40 in one case and 0.70 in the other; Table 5). In contrast, the individual horse did not seem to explain much of the variation in behaviours related to locomotion and the pinning of the ears backwards (ie the value of the ICC is less than 0.40; Table 5).

### Correlation

Lunge tension was correlated with right tension (0.37, p-value < 0.001), total tension (0.19, p-value < 0.05) and asymmetry coefficient (0.52, p-value < 0.0001). Opening the mouth was correlated with total, right and left tension (respectively 0.25, p-value = 0.002; 0.24, p-value = 0.003; 0.24, p-value = 0.004).

## Discussion

Total RMS tension ranged from 6.2 to 11.7 kg with a right RMS tension superior to left RMS tension. When tension was high, mouth opening increased. The total rein tension and the number of mouth opening were the lowest with the draw reins. Mouth opening was thus thought to be a behavioural sign of discomfort [28], which increased with short reins [25] and high rein tensions [18].

### Rein tension values

Regular contact is essential, but the optimal intensity remains to be defined. The tension of reins with a horse in hand can be a reference for the rider, which must not be exceeded [25]. They described a comfortable tension of less than 10 N while respecting the vertical position of the nose line, knowing that the highest mean voluntary acceptance of rein tensions for young horses was 20 N in total [25] and the maximum voluntary rein tension that occurred was 43.9 N with ponies and cold-blood horses, and 29 N with warmblood horses [18]. The rein tension values in this present study were similar to the values in the ridden condition [18, 21] Moreover, R^2^ conditional values showed that 76% of total tension variability was explained by rein type and "the horse" and 20% by rein type alone. In this context of measurement with international-level vaulting horses, the horse’s contribution to tension values was higher than that described with data collected from regular ridden training sessions [21, 26]. It remains tricky to compare the tension values obtained with those in the literature, given all the factors that affect them, particularly the discipline or type of exercise. The values obtained in this study show the effect of the latter, which in this research was the left canter on a 15-metre circle "with the impression of carrying out the Test of its own accord and staying in true balance and self-carriage, with a picture of harmony and lightness throughout the Test" (definition of Vaultability by the FEI). The question is whether these requirements can be met by lower tension values than those obtained in this present study. The vertical nose line obtained through the reins is not the only criterion of quality, as it is also a matter of obtaining engagement of the whole body of the horse. The FEI specifies that the aim of training is to further develop and improve the horse’s balance and to develop and increase the horse’s ability to engage its hindquarters. Reins affect the position of the head and neck, two segments that influence the horse’s kinematics [3], in particular the kinetics and kinematics of fore and hind limbs, as well as thoracolumbar movement [3, 4, 32–34]. Yet, similar kinematic changes can produce different kinetic patterns [35]. Reducing the head-neck angle is not enough to obtain the desired engagement and collection. According to Biau et al. [36], altering head and neck position through various types of reins mainly affects the kinetics of the forelimb. In this previously study, Rubber bands, Chambons and Back lift significantly increased forelimb propulsion at trot, and only one increased the activity of hindlimbs. A study has shown that the draw rein in combination with normal reins can increase weight-bearing and propulsion in the hind limbs [37]. However, this concerned the ridden condition. In the present study, horses could have chosen to avoid this pressure on the mouth (or nose in the case of the NOTM), then they would have shown a nose beyond the vertical. Perhaps these horses needed a “support” for this collected vaulting canter. The horse push against the bit [23]. Yet, the equestrian describe the keeping of collection without rider contact “rendre la main” or “descente de main” [38, 39]. But the tension peaks linked to the strides are unavoidable [22, 23], especially as the reins are fixed on the surcingle. To respect the physical integrity of the mouth, the challenge would be to reduce these peak values while keeping a minimum of constant contact. Further analysis of the locomotion is essential in order to completely describe the tension per stride, body position and engagement that is expected to support the vaulter, while restricting movements of the head and neck in order to be able to perform safely.

The tensions on the outside of the circle were higher than those measured on the inside of the circle. The rein tensions on a circle with a rider are also not symmetrical, as the effect of the exercise is mixed with an effect of the rider’s laterality [40]. In this study, the asymmetry was directly linked to the horse’s kinematic movements on the circle. Changes of the neck angle occur before those of the trunk [32], which explains that when the horse is moving on a circle to the left, there is a higher right-rein peak tension, because the distance between the two points of attachment of the rein is constant; this is in contrast to when a horse is ridden and the rider can move their hand forward at the time of the peak to reduce the value of the tension. The tip of the nose moves to the left just before the head is extended, when the trunk is still flexed (Fig in S3 File). For ASYMMETRY, the marginal R^2^ value (0.14) was lower than those for TOTAL (0.20), and LEFT (0.27) indicating that ASYMMETRY was slightly more dependent on factors other than rein type than were TOTAL and LEFT tensions. Moreover, the addition of the "individual horse" effect explains only 52% of the variability in the ASYMMETRY variable, compared to 70–75% for LEFT, RIGHT and TOTAL tensions. Thus, the lunger also probably contributes to this asymmetry, bringing the tip of the nose towards the inside of the circle, thus increasing external tension. This hypothesis is corroborated by the significant correlation between the right rein tension, the asymmetry and the lungeline tension. The difference in length between the right and left reins, which was left to the lunger to decide, undoubtedly has an impact on this asymmetry as well.

### Effect of rein type

In this study, rein tensions were compared between five rein types. The conditional and marginal R^2^ values attested to the relevance of the effect of rein type on the tension values. 76% of the variability in total tension values was explained by the reins and the horse, and 20% by the type of reins. As expected, total tension recorded with the draw reins were significantly lower than those recorded with the side reins. The draw rein with "upper triangle" adjustment seems to be a good compromise between the five types of reins in terms of tension, since it demonstrated the lowest tensions. It would help to reduce maximum tension values by providing limited movement around the ring, whereas the side reins impose a limit. Moreover, this type of rein was significantly associated with the least expression of mouth openings and head movements. However, the statistical models clearly showed that the behaviours reflecting discomfort were more related to others factors than to rein type. Indeed only 1 to 4% of the variability of behaviours was explained by the type of reins. Regarding tail swishing, head movements, mouth opening and abnormal tail position, it seems that there is a moderate to strong individual effect. However, behaviours related to locomotion and the expression of ears pointed backward seem to be related to others factors than the rein type and the individual horse, for example the environment in which the test was carried out.

One study described a decrease in the maximum peak with elastics but also an increase in minimum values [23]. This could explain why there are no differences between the three types of side reins in this study insofar as our type of calculation of mean tension values reflects both maximum and minimum peaks as well as their duration. Unlike the ridden condition, where the peaks are thought to be more linked to the horse and the minimum values to the rider’s action, in hand, with surcingle fixed to the trunk, the values are totally linked to the horse’s kinematics, with a potential impact of lunger. Some elastic products claim to "help the rider create an independent hand with a soft, forward contact" [41], to protect the horse’s mouth from rider error. However, Randle and al [42] found that although elastic reins resulted in lower levels of rein tension than non-elastic reins during normal riding, the peak rein tension applied during the transition to stop was significantly higher. In addition, elastic reins release tension more slowly and with many more fluctuations observed towards zero. This can hinder learning process by reducing the clarity of the aid and delaying the reinforcement of correct behaviour due to the inability to release bit pressure at the same time as the desired reaction. This is consistent with the findings of Heleski et al. [43] that elastic rein inserts did not reduce the number of discomfort behaviours in horses ridden by novices. In the present study, the number of HEAD behaviours was higher with all-elastic side reins but with a significant difference only with draw reins s and low (4% of the behavioural variability). Moreover, MOUTH was more expressed with all-leather side reins without elastic but with a significant difference only with draw reins. The marginal R^2^ values (0.04 and 0.03 respectively for HEAD and MOUTH) minimized this elastic effect.

The values for MOUTH and ABN TAIL behaviours were particularly high compared to those of the bibliography [16]. This can be explained by the fact that the end of the rein was fixed to the surcingle, unlike the rider’s hand, which moves. Total tension and RIGHT tension were correlated with the expression of the MOUTH behaviour: the greater the tension, the more the open mouth indicator was expressed.

The NOM was clearly different from other types of reins. Right and left tensions were higher than those measured with the others rein types. Symmetry was almost achieved, and the tension of the lunge line was lower. Even though the lunger contributed to bringing the tip of the nose inwards, and although the tension on the lunge line was lower, this did not impact the external tension as much as with the other rein types, since the point of attachment was on the noseband, and higher than the tip of the nose. This rein type certainly freed up the mouth, and the number of mouth behaviours was lower than with the side reins without elastic. However, very high tensions can have harmful effects such as those described in the case of a noseband that is too tight, neural damage, alteration of blood circulation by compression of the vessels in the head, tissue damage, and restriction on breathing [44]. These damages described in the bibliography are linked to the action of the man tightening the noseband. This is not the case in our study, it is the horse itself that is pulling. The pressure on the noseband is perhaps less painful than that of the mouth, which explains the Mouth behaviours lower than the side reins without elastic, despite the fact that many horses discovered this on the day of the tests.

### Limitations

A limitation to this study was that some horses were not accustomed to using certain types of reins. This was the case with the NOM. Even with a period of adaptation before the measurements were taken, the horse may have shown high rein tensions due to being unaccustomed to the equipment. The lunger in this context was also able to modify their contact but the lunge line tension was not significantly different between rein types.

To respect the training habits of these high-level couples, the difference in length between right and left reins has not been imposed, which inevitably impacted results. However, the aim of this study was to assess the state of tension in international-level horses, so enforcing an adjustment and potentially changing training habits could also have constituted a bias, and the low standard deviation of the assymmetry coefficient showed that there was a consensus among lungers on the adjustment of this difference in right and left reins length depending on the horse.

Another limitation was that the measurement conditions were not entirely similar. In fact, despite the specifications provided upstream to the organizer of the measurement session, certain parameters were not completely controllable. This was the case for the quality of the surface, where even if the components are identical, the state of maintenance can have an impact on the surface properties. For example, a loose surface can cause an increase in the workload of a vaulting horse [45] and potentially, therefore, intensify discomfort.

## Conclusion

Measuring tensions and evaluating signs of discomfort for several rein types allowed us to identify one that helped reduce tensions and several behavioural signs of discomfort such as head movements and mouth openings, even though the effect of the horse was predominant. Draw reins with an upper adjustment triangle seems a good compromise to perform while respecting vaulting horse comfort. Indeed with this rein type, the number of mouth behaviour and tensions were significantly lower than those measured with the side rein types with or without elastic, fixed on the bit ring or the noseband. These results are important for guiding FEI Vaulting administrators in the formulation of rules and to help lungers choose the adapted type of rein and its adjustment, which will contribute to better performance while respecting the physical and mental integrity of the horse. Making the best choice of rein type for the work of these international-level vaulting horses will contribute to improving comfort by reducing tension, but other factors not taken into account in this study should be considered.

## Supporting information

S1 File Additional information on the calculation of mixed models. (DOCX)

S2 File Calculation of draw rein tensions. (DOCX)

S3 File Nose inclination. (DOCX)

S1 Data (CSV)

S2 Data (XLSX)
