# Supplementary material for: Rein tensions and behaviour with five rein types in international-level vaulting horses
Source: PLoS One. 2024 Oct 17;19(10):e0311919. doi: 10.1371/journal.pone.0311919 (PMC11486377; doi:10.1371/journal.pone.0311919)
Supplement: S1 File — (DOCX) [file pone.0311919.s001.docx]

Supplementary file

This is the Title of S1 file: additional information on the calculation of mixed models

| sqrt(LEFT) ~ type of reins + (1\|horse) | | | | | |
| --- | --- | --- | --- | --- | --- |
| **Fixed effects** | Est/Beta | SE | 95% CI | t | p |
| Intercept | 53.4 | 2.4 | 48.6 – 58.1 | 22.1 | <0.001 |
| All elastic | -0.9 | 2.0 | -4.8 – 3.1 | -0.4 | 0.67 |
| Without elastic | -1.1 | 2.0 | -5.1 – 2.9 | -0.5 | 0.60 |
| Upper triangle | -6.6 | 2.0 | -10.6 - -2.6 | -3.2 | <0.01 |
| Not on the mouth | 21.4 | 2.2 | 17.1 – 25.6 | 9.7 | <0.001 |
| **Random effects** | Variance | | SD |  |  |
| Horse (Intercept) | 150.1 | | 12.2 |  |  |
| **Model fit** | R^2^ Marginal | | R^2^ Conditional |  |  |
|  | 0.27 | | 0.77 |  |  |

| sqrt(RIGHT) ~ type of reins + (1\|horse) | | | | | |
| --- | --- | --- | --- | --- | --- |
| **Fixed effects** | Est/Beta | SE | 95% CI | t | p |
| Intercept | 64.1 | 2.5 | 59.2 – 68.9 | 25.9 | <0.001 |
| All elastic | -1.7 | 2.2 | -6.0 – 2.7 | -0.7 | 0.46 |
| Without elastic | 1.4 | 2.2 | -2.9 – 5.8 | 0.6 | 0.53 |
| Upper triangle | -4.3 | 2.2 | -8.6 – 0.1 | -1.9 | 0.06 |
| Not on the mouth | 11.3 | 2.4 | 6.7 – 16.0 | 4.7 | <0.001 |
| **Random effects** | Variance | | SD |  |  |
| Horse (Intercept) | 145.0 | | 12.0 |  |  |
| **Model fit** | R^2^ Marginal | | R^2^ Conditional |  |  |
|  | 0.10 | | 0.67 |  |  |

| sqrt(TOTAL) ~ type of reins + (1\|horse) | | | | | |
| --- | --- | --- | --- | --- | --- |
| **Fixed effects** | Est/Beta | SE | 95% CI | t | p |
| Intercept | 83.6 | 3.2 | 77.4 – 89.9 | 26.3 | <0.001 |
| All elastic | -1.7 | 2.6 | -6.8 – 3.3 | -0.7 | 0.51 |
| Without elastic | 0.5 | 2.6 | -4.6 – 5.6 | 0.2 | 0.85 |
| Upper triangle | -7.2 | 2.6 | -12.3 - -2.11 | -2.7 | <0.01 |
| Not on the mouth | 23.2 | 2.8 | 17.7 – 28.6 | 8.2 | <0.001 |
| **Random effects** | Variance | | SD |  |  |
| Horse (Intercept) | 265.7 | | 16.3 |  |  |
| **Model fit** | R^2^ Marginal | | R^2^ Conditional |  |  |
|  | 0.20 | | 0.76 |  |  |

| log(ASYMMETRY) ~ type of reins + (1\|horse) | | | | | |
| --- | --- | --- | --- | --- | --- |
| **Fixed effects** | Est/Beta | SE | 95% CI | t | p |
| Intercept | 0.4 | 0.1 | 77.8 – 89.7 | 5.5 | <0.001 |
| All elastic | -0.0 | 0.1 | -6.9 – 3.1 | --0.1 | 0.95 |
| Without elastic | 0.5 | 0.1 | -4.5 – 5.6 | 0.7 | 0.49 |
| Upper triangle | 0.1 | 0.1 | -33.5 - -23.4 | 1.9 | 0.06 |
| Not on the mouth | -0.4 | 0.1 | 17.5 – 28.3 | -4.6 | <0.001 |
| **Random effects** | Variance | | SD |  |  |
| Horse (Intercept) | 0.08 | | 0.3 |  |  |
| **Model fit** | R^2^ Marginal | | R^2^ Conditional |  |  |
|  | 0.14 | | 0.52 |  |  |

| sqrt(LUNGE) ~ type of reins + (1\|horse) | | | | | |
| --- | --- | --- | --- | --- | --- |
| **Fixed effects** | Est/Beta | SE | 95% CI | t | p |
| Intercept | 54.6 | 2.8 | 77.8 – 89.7 | 19.5 | <0.001 |
| All elastic | 4.1 | 2.1 | -6.9 – 3.1 | 1.9 | 0.06 |
| Without elastic | 4.7 | 2.1 | -4.5 – 5.6 | 2.2 | 0.03 |
| Upper triangle | 3.2 | 2.1 | -33.5 - -23.4 | 1.5 | 0.13 |
| Not on the mouth | -3.8 | 2.1 | 17.5 – 28.3 | -1.8 | 0.08 |
| **Random effects** | Variance | | SD |  |  |
| Horse (Intercept) | 211.9 | | 14.5 |  |  |
| **Model fit** | R^2^ Marginal | | R^2^ Conditional |  |  |
|  | 0.03 | | 0.76 |  |  |

| sqrt(LOC) ~ type of reins + (1\|horse) | | | | | |
| --- | --- | --- | --- | --- | --- |
| **Fixed effects** | Est/Beta | SE | 95% CI | t | p |
| Intercept | 0.8 | 0.1 | 0.5 – 1.1 | 5.6 | <0.001 |
| All elastic | 0.1 | 0.2 | -0.2 – 0.5 | 0.7 | 0.45 |
| Without elastic | 0.2 | 0.2 | -0.1 – 0.5 | 1.3 | 0.21 |
| Upper triangle | 0.3 | 0.2 | -0.1 – 0.6 | 1.8 | 0.07 |
| Not on the mouth | 0.2 | 0.2 | -0.2 – 0.5 | 0.9 | 0.32 |
| **Random effects** | Variance | | SD |  |  |
| Horse (Intercept) | 0.2 | | 0.5 |  |  |
| **Model fit** | R^2^ Marginal | | R^2^ Conditional |  |  |
|  | 0.01 | | 0.35 |  |  |

| TAIL ~ type of reins + (1\|horse) | | | | | |
| --- | --- | --- | --- | --- | --- |
| **Fixed effects** | Est/Beta | SE | 95% CI | t | p |
| Intercept | -2.1 | 0.6 | -3.3 - -1.0 | -3.7 | <0.001 |
| All elastic | 0.1 | 0.3 | -0.4 – 0.7 | 0.6 | 0.57 |
| Without elastic | 0.4 | 0.2 | -0.1 – 0.8 | 1.4 | 0.16 |
| Upper triangle | 0.03 | 0.3 | -0.5 – 0.6 | 0.1 | 0.9 |
| Not on the mouth | -0.2 | 0.3 | -0.8 – 0.3 | -0.8 | 0.41 |
| **Random effects** | Variance | | SD |  |  |
| Horse (Intercept) | 5.3 | | 2.3 |  |  |
| **Model fit** | R^2^ Marginal | | R^2^ Conditional |  |  |
|  | 0.01 | | 0.72 |  |  |

| HEAD ~ type of reins + (1\|horse) | | | | | |
| --- | --- | --- | --- | --- | --- |
| **Fixed effects** | Est/Beta | SE | 95% CI | t | p |
| Intercept | -2.3 | 0.8 | -3.8 - -0.7 | -2.8 | 0.004 |
| All elastic | 1.1 | 0.5 | 0.1 – 2.0 | 2.2 | 0.03 |
| Without elastic | -0.4 | 0.6 | -1.5 – 0.7 | -0.7 | 0.51 |
| Upper triangle | -0.8 | 0.6 | -2.1 – 0.4 | -1.4 | 0.17 |
| Not on the mouth | -0.3 | 0.6 | -1.5 – 0.9 | -0.5 | 0.62 |
| **Random effects** | Variance | | SD |  |  |
| Horse (Intercept) | 6.2 | | 2.5 |  |  |
| **Model fit** | R^2^ Marginal | | R^2^ Conditional |  |  |
|  | 0.04 | | 0.71 |  |  |

| sqrt(MOUTH) ~ type of reins + (1\|horse) | | | | | |
| --- | --- | --- | --- | --- | --- |
| **Fixed effects** | Est/Beta | SE | 95% CI | t | p |
| Intercept | 4.2 | 0.4 | 3.5 – 5.4 | 9.3 | <0.001 |
| All elastic | -0.7 | 0.4 | -1.5 – 0.1 | -1.6 | 0.10 |
| Without elastic | 0.1 | 0.4 | -0.7 – 0.9 | 0.1 | 0.93 |
| Upper triangle | -1.3 | 0.4 | -2.1 – -0.5 | -3.1 | 0.002 |
| Not on the mouth | -0.9 | 0.4 | -1.7 – -0.1 | -2.0 | 0.04 |
| **Random effects** | Variance | | SD |  |  |
| Horse (Intercept) | 5.1 | | 2.2 |  |  |
| **Model fit** | R^2^ Marginal | | R^2^ Conditional |  |  |
|  | 0.03 | | 0.64 |  |  |

| EARS ~ type of reins + (1\|horse) | | | | | |
| --- | --- | --- | --- | --- | --- |
| **Fixed effects** | Est/Beta | SE | 95% CI | t | p |
| Intercept | 2.4 | 0.2 | 1.9 – 2.8 | 9.7 | <0.001 |
| All elastic | 0.1 | 0.3 | -0.5 – 0.6 | 0.1 | 0.88 |
| Without elastic | -0.1 | 0.3 | -0.7 – 0.5 | -0.3 | 0.78 |
| Upper triangle | -0.4 | 0.3 | -0.9 – 0.2 | -1.2 | 0.25 |
| Not on the mouth | -0.2 | 0.3 | -0.8 – 0.4 | -0.6 | 0.57 |
| **Random effects** | Variance | | SD |  |  |
| Horse (Intercept) | 0.4 | | 0.6 |  |  |
| **Model fit** | R^2^ Marginal | | R^2^ Conditional |  |  |
|  | 0.01 | | 0.27 |  |  |

| ABN TAIL ~ type of reins + (1\|horse) | | | | | |
| --- | --- | --- | --- | --- | --- |
| **Fixed effects** | Est/Beta | SE | 95% CI | t | p |
| Intercept | 65.9 | 5.6 | 54.9 – 76.8 | 11.7 | <0.001 |
| All elastic | -7.3 | 6.1 | -19.3 – 4.6 | -1.2 | 0.24 |
| Without elastic | 0.5 | 6.2 | -11.5 – 12.6 | 0.1 | 0.93 |
| Upper triangle | -6.9 | 6.1 | -18.7 – 4.8 | -1.1 | 0.25 |
| Not on the mouth | -2.4 | 6.3 | -14.6 – 9.8 | -0.4 | 0.70 |
| **Random effects** | Variance | | SD |  |  |
| Horse (Intercept) | 526.4 | | 22.9 |  |  |
| **Model fit** | R^2^ Marginal | | R^2^ Conditional |  |  |
|  | 0.01 | | 0.45 |  |  |
