# Supplementary material for: Rein tensions and behaviour with five rein types in international-level vaulting horses
Source: PLoS One. 2024 Oct 17;19(10):e0311919. doi: 10.1371/journal.pone.0311919 (PMC11486377; doi:10.1371/journal.pone.0311919)
Supplement: S2 File — (DOCX) [file pone.0311919.s002.docx]

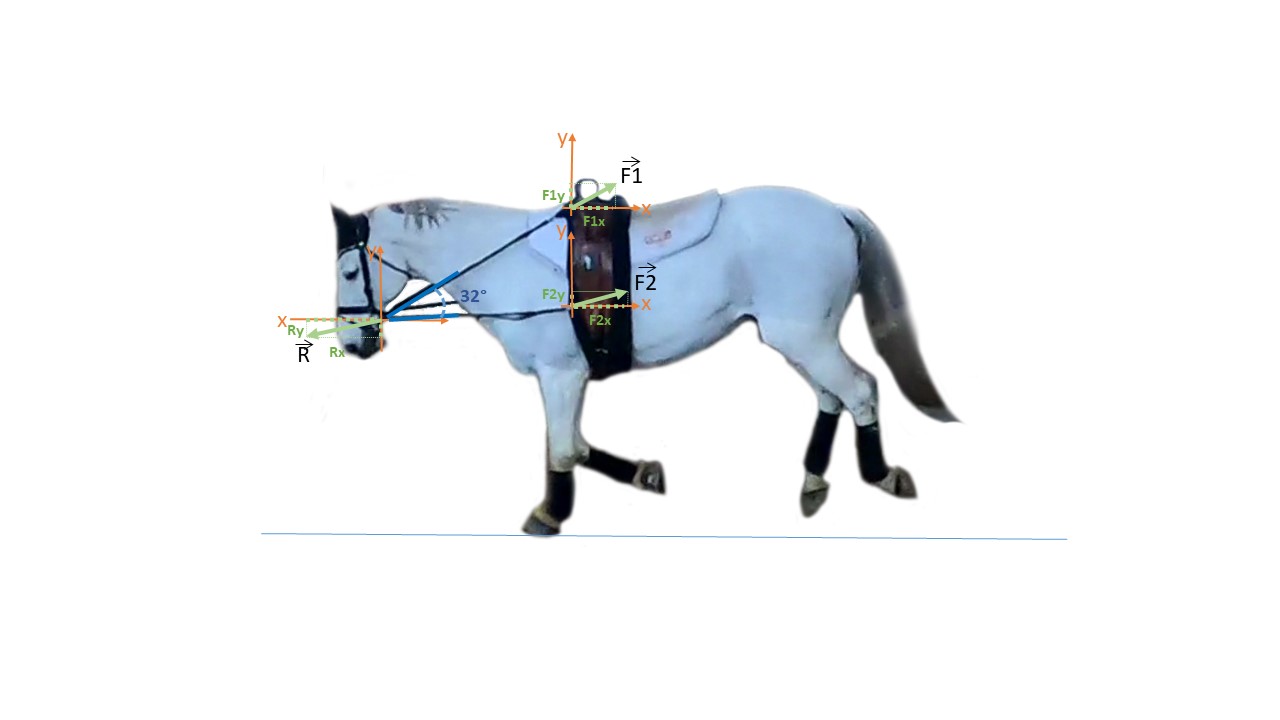
Supplementary file

**This is the S2 file Title: Calculation of Draw rein tensions**

$\vec{\boldsymbol{F}\boldsymbol{1}}\boldsymbol{+}\vec{\boldsymbol{F}\boldsymbol{2}}\boldsymbol{=}\vec{\boldsymbol{R}}$

Projection on the x (horizontal) and y (vertical) axes

α= angle between x axis and $\vec{F1}$

β=angle between x axis and $\vec{F2}$

F1x = F1 cos α

F1y = F1 sin α

α

β

F2x = F2 cos β

F2y = F2 sin β

Rx=F1x+F2x

Ry=F1y+F2y

Rx=F1cosα+F2cosβ

Ry=F1sinα+F2sinβ

The reins were considered to be taut : **F1 = F2 = F**

$$R=\sqrt{R^{2}x+R^{2}y}$$

$$R=\sqrt{(F1cos\alpha+F2cos\beta)^{2}+(F1sin\alpha+F2sin\beta)^{2}}$$

In thi picture case, θ= α - β

$$R=\sqrt{2}*F*\sqrt{1+\cos(\theta)}$$

Avec $\theta$=32°, **R=F*1.9**
