# Supplementary material for: Rein tensions and behaviour with five rein types in international-level vaulting horses
Source: PLoS One. 2024 Oct 17;19(10):e0311919. doi: 10.1371/journal.pone.0311919 (PMC11486377; doi:10.1371/journal.pone.0311919)
Supplement: S3 File — (DOCX) [file pone.0311919.s003.docx]

Supplementary file

**This is the Title of S3 file : Nose inclination**


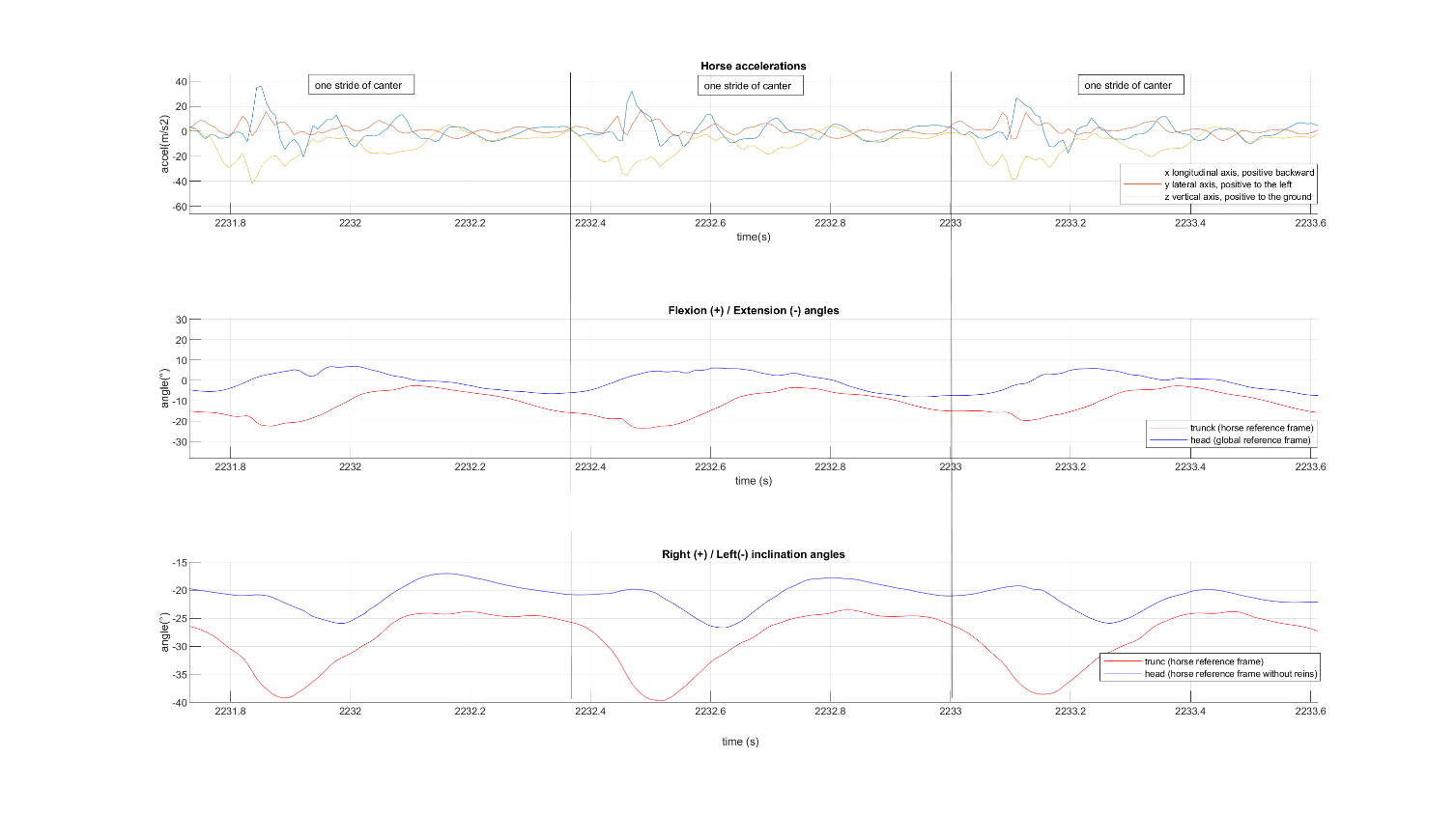


**Figure 1: above, horse’s truck accelerations (m/s²) of three stride of left canter; center : flexion/ extension angles (°) of the head (blue) and of the trunc (red) ; blow : inclination angles (°) of the head (blue) and trunck (red).**

The angles were calculated from the IMUs (Opal, APDM Inc., Portland, OR, USA) fixed against the sternum for the trunck and attached to the snaffle for the head.

The tip of the nose moves to the left just before the head was extended, when the trunk was still flexed
